# Frequency-based brain networks: From a multiplex framework to a full multilayer description

**DOI:** 10.1162/netn_a_00033

**Published:** 2018-10-01

**Authors:** Javier M. Buldú, Mason A. Porter

**Affiliations:** Laboratory of Biological Networks, Center for Biomedical Technology (UPM), Pozuelo de Alarcón, Madrid, Spain; Complex Systems Group & G.I.S.C., Universidad Rey Juan Carlos, Móstoles, Madrid, Spain; Department of Mathematics, University of California Los Angeles, Los Angeles, CA, USA; Oxford Centre for Industrial and Applied Mathematics, Mathematical Institute, University of Oxford, Oxford, UK; CABDyN Complexity Centre, University of Oxford, Oxford, UK

**Keywords:** Functional brain networks, Magnetoencephalography, Multilayer networks, Multiplex networks, Algebraic connectivity

## Abstract

We explore how to study dynamical interactions between brain regions by using functional multilayer networks whose layers represent different frequency bands at which a brain operates. Specifically, we investigate the consequences of considering the brain as (i) a multilayer network, in which all brain regions can interact with each other at different frequency bands; and as (ii) a multiplex network, in which interactions between different frequency bands are allowed only within each brain region and not between them. We study the second-smallest eigenvalue *λ*_2_ of the combinatorial supra-Laplacian matrix of both the multiplex and multilayer networks, as *λ*_2_ has been used previously as an indicator of network synchronizability and as a biomarker for several brain diseases. We show that the heterogeneity of interlayer edge weights and, especially, the fraction of missing edges crucially modify the value of *λ*_2_, and we illustrate our results with both synthetic network models and real data obtained from resting-state magnetoencephalography. Our work highlights the differences between using a multiplex approach and a full multilayer approach when studying frequency-based multilayer brain networks.

## Author Summary

For more than a decade, network analysis has been used to investigate the organization and function of the human brain. However, applications of multilayer network analysis to neuronal networks are still at a preliminary stage, in part because of the difficulties of adequately representing brain-imaging data in the form of multilayer networks. In this study, we investigate the main differences in using multiplex networks versus more general multilayer networks when constructing frequency-based brain networks. Specifically, we are concerned with the differences for estimating the algebraic connectivity *λ*_2_, which has been related to structural, diffusion, and synchronization properties of networks. Using synthetic network models and real data, we show how edge-weight heterogeneity and missing interlayer edges crucially influence the value of *λ*_2_.

## INTRODUCTION

During the last few years, network science has undergone a conceptual revolution with the extension of well-established techniques of network analysis to multilayer brain networks (Boccaletti et al., 2014; De Domenico et al., 2013; Kivelä et al., 2014), which provide a convenient way to simultaneously encode different types of interactions, subsystems, and other complications in networks. Consequently, it has been necessary to revisit our intuitive understanding of both structural and dynamical properties of networks—including structural phase transitions (Radicchi & Arenas, 2013), diffusion and other spreading processes (De Domenico, Granell, Porter, & Arenas, 2016; Gómez et al., 2013; Salehi et al., 2015), percolation and robustness (Buldyrev, Parshani, Paul, Stanley, & Havlin, 2010; Gao, Buldyrev, Stanley, Xu, & Havlin, 2013), synchronization (Aguirre, Sevilla-Escoboza, Gutiérrez, Papo, & Buldú, 2014), and others—to the new possibilities in multilayer descriptions, leading in many cases to counterintuitive results.

**Figure 1.**
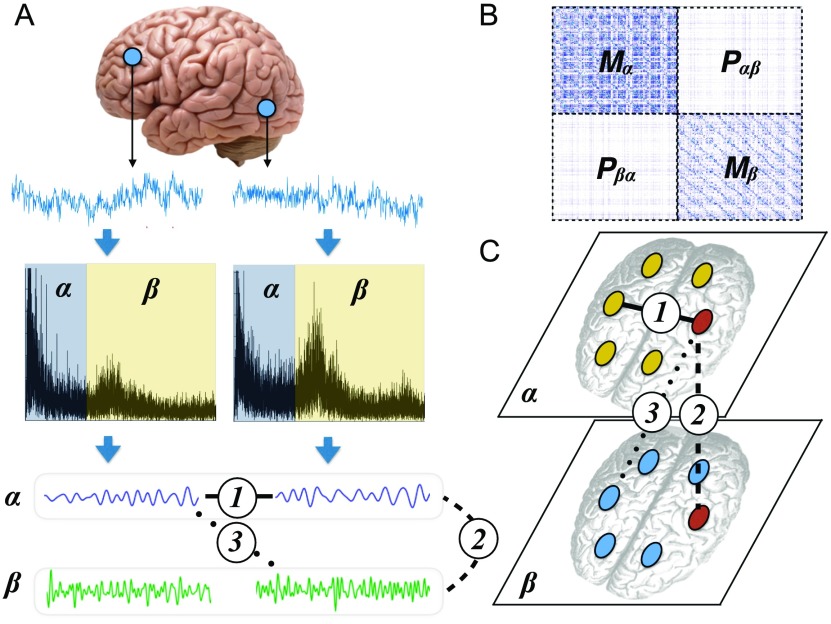
Encoding brain dynamics as a multilayer functional network. We show an illustrative example with two frequency bands (alpha and beta). (A) We band-pass filter the MEG signals at two frequency bands: alpha [8–12] Hz and beta [12–30] Hz. We use mutual information (MI; MacKay, 2003) to quantify coordination between brain regions. This yields three different type of functional edges: Edge type “1” quantifies coordination between different regions at the same frequency band; edge type “2” corresponds to interlayer edges, which couple the activity of the same region at different frequency bands; and edge type “3” quantifies cross-frequency coupling (CFC) between two brain regions. Multiplex networks include only edges of types 1 and 2, whereas more general multilayer networks include all three types of edges. (B) Schematic of the supra-adjacency matrix of a two-layer network constructed from the data in panel A. (C) Schematic of the intralayer and interlayer edges in the multilayer functional network.

**Figure 2.**
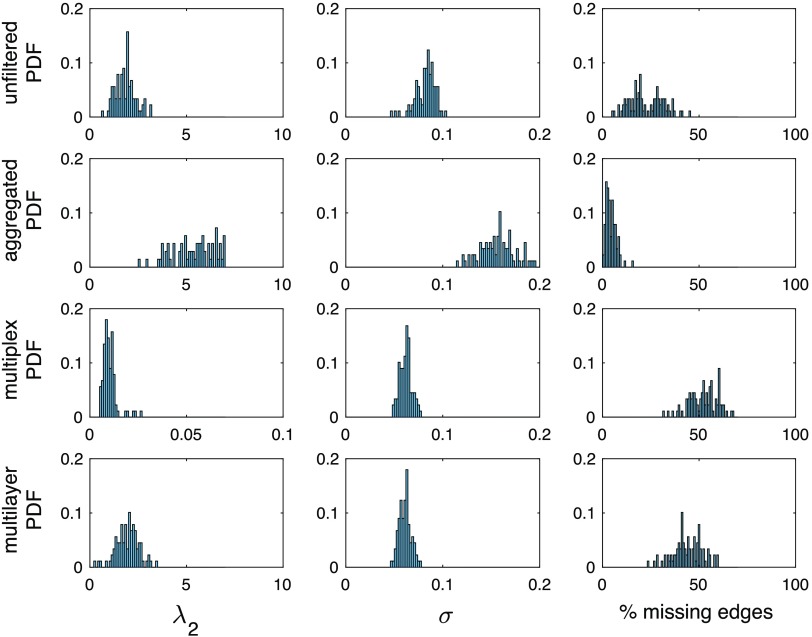
MEG data: from unfiltered data to a multilayer network. Probability density functions (PDFs) of different network characteristics of a group of *q* = 89 individuals. (See Materials and Methods for details.) We show the second-smallest eigenvalue *λ*_2_ of the combinatorial Laplacian matrix, the standard deviation *σ* of the matrix elements (to quantify their heterogeneity), and the percentage of missing edges of four different networks: (i) the functional network obtained from the unfiltered signals (first row), (ii) an aggregated network of the alpha and beta layers (second row), (iii) a multiplex network (third row), and a (iv) full multilayer network (fourth row). In all cases, we only consider two layers (alpha and beta). For the multiplex and multilayer networks, note that we do these computations with the combinatorial supra-Laplacian matrices. The percentage of missing edges in the unfiltered and aggregated networks is equal to the percentage of 0 values in the whole matrix, but it refers only to the interlayer edges for the multiplex and multilayer networks.

The study of brain networks is currently undergoing a process of adaptation of classical single-layer (“monolayer”) concepts and analyses to a more general multilayer description. (For reviews, see Betzel & Bassett, 2016; De Domenico, 2017; Vaiana & Muldoon, 2018; also see Figure 1 of Betzel & Bassett, 2016 and Figures 1–2 of De Domenico, 2017 for relevant schematics.) Some studies have considered integration of data from structural and functional brain imaging into a multilayer network to account for both anatomical and dynamical information. In an early study using monolayer networks, Honey, Koötter, Breakspear, and Sporns (2007) showed that a dynamical model simulated on the anatomical network of a macaque neocortex can successfully identify the positions of the anatomical hubs when signals are averaged appropriately. More recently, Stam et al. (2016) analyzed how anatomical networks support activity, leading to specific functional networks (either undirected ones or directed ones), demonstrating that a dynamical model close to a critical transition is able to unveil interactions between structural and functional networks.

It is also possible to combine anatomical and functional interactions of just a few nodes instead of an entire brain network. For example, Battiston, Nicosia, Chavez, and Latora (2017) examined network motifs (i.e., overrepresented network substructures that consist of a few nodes; Milo et al., 2002) that combine anatomical connections (in one layer) and functional relations between cortical regions (in a second layer), linking data obtained, respectively, from diffusion-weighted magnetic resonance imaging (DW–MRI) and functional magnetic resonance imaging (fMRI).

Efforts to combine anatomical and functional networks into a single multilayer network face the challenge of how to normalize the weights of edges that arise from different origins. To tackle this issue, Simas, Chavez, Rodriguez, and Díaz-Guilera (2015) proposed translating functional and anatomical networks into a common embedding space and then comparing them in that space. They constructed functional networks (each with *N* nodes) from the fMRIs of *q* healthy individuals. They then used the functional networks of the *q* = 20 individuals to construct a single functional multiplex brain network, a special type of multilayer network in which corresponding entities (brain regions) in different layers (individuals) can be connected to each other via interlayer edges, but other types of interlayer edges cannot occur (Kivelä et al., 2014). They followed a similar procedure to construct an anatomical multiplex network using data obtained from DW–MRI. They then independently projected each of the multiplex networks into a common embedding space using a series of algebraic operations that allow one to calculate an “algebraic aggregation” of all layers into a single layer (see Simas et al., 2015, for details). Using such a projection, it is possible to quantify the differences between anatomical and functional networks. Simas et al. also calculated an “averaged aggregation” for each of the functional and anatomical multiplex networks by averaging the weights of the corresponding edges over all layers. They compared the two types of aggregation, and they were thereby able to identify certain brain regions (e.g., ones related to visual, auditory, and self-awareness processes) with significant differences between the functional and anatomical networks for both types of aggregation. However, only the algebraic aggregation was able to detect differences between the functional and anatomical networks in other regions (specifically, the thalamus, the amygdala, the postcentral gyrus, and the posterior cingulate), suggesting that the averaged aggregation disregards significant information (Simas et al., 2015).

One possible alternative for reducing the complexity of analysis of brain networks is to concentrate only on “functional” (dynamical, in fact) interactions between brain regions and to define multilayer functional networks as a concatenation of a series of layers, each of which captures the interplay between brain regions during some time window. This approach, in which a layer in a multilayer network represents connection similarities over some time window, was taken in papers such as Bassett et al. (2011, 2013) and Braun et al. (2014) to analyze the temporal evolution of network modules and examine dynamical reconfiguration and “flexibility” of functional networks.

Another alternative is to construct functional multilayer networks whose layers correspond to the well-known frequency bands at which a brain operates (Buzsáki, 2006). As demonstrated by Brookes et al. (2016), it is possible to construct frequency-based multilayer networks from magnetoencephalographic (MEG) recordings by (i) band-pass filtering the raw MEG signals, (ii) obtaining the envelope of the amplitude at each frequency band, and (iii) measuring the pairwise correlations between each envelope (for whichever frequency it accounts). Using such an approach, Brookes et al. (2016) constructed frequency-based multilayer networks, in which each layer includes the interactions in a given frequency band, and showed that the corresponding supra-adjacency matrices (which encode a linear-algebraic representation of connections in a multilayer network) convey statistically significant differences when comparing a control group with a group of schizophrenia sufferers.

Recently, De Domenico, Sasai, and Arenas (2016) took the important step of analyzing the spectral properties of matrices associated with frequency-based multiplex networks. The spectrum of an Ñ×Ñ matrix consists of the set $\left\{\lambda_{1},\lambda_{2},\lambda_{3},\ldots ,\lambda_{Ñ}\right\}$ of its eigenvalues, and it encodes valuable information about the structural properties of the corresponding network. In turn, these eigenvalues (as we will explain later) are related to various dynamical properties, such as network synchronizability, robustness, and diffusion (Newman, 2010). De Domenico, Sasai, and Arenas (2016) compared a group of schizophrenic patients with a control group by using fMRI data, and they found that the second-smallest eigenvalue (i.e., the algebraic connectivity or “Fiedler value”; Van Mieghem, 2011) *λ*_2_ of the combinatorial supra-Laplacian matrix^1^ associated with a multiplex network is a better discriminator between the two groups than what one can obtain by studying either unfiltered or single-band functional networks (i.e., by using monolayer networks). De Domenico et al. also calculated centrality measures (i.e., measures of the importance of network components; Newman, 2010) on the frequency-based multiplex networks to demonstrate the existence of hubs that had not been classified previously as important brain regions for functional integration. Hubs of the control group were located in anterior cingulate, superior frontal, insula, and superior temporal cortices; however, hubs for schizophrenic patients were distributed over frontal, parietal, and occipital cortices. These results revealed that frequency-based multiplex networks include relevant information about the functional organization of brain networks that is not captured by using a classical monolayer approach.

Multiple recent papers have demonstrated the benefits of using a multiplex description for analyzing the functional networks of patients suffering from Alzheimer’s disease (AD). For example, Yu et al. (2017) constructed frequency-based multiplex networks from MEG data and demonstrated that centrality measures calculated with layers analyzed independently are unable to detect significant differences between a control group and the AD group. However, when centralities are evaluated on a frequency-based multiplex network, one can find statistically significant differences in the hippocampus, posterior default-mode network, and occipital areas. Guillon et al. (2017) also used frequency-based multiplex networks to differentiate between controls and people with AD. In this case, the authors proposed the use of a multi-participation coefficient (MPC) to enhance classification of which individuals are suffering from AD. Their MPC consists of an adaptation of the classical (i.e., monolayer) participation coefficient (Guimerà & Amaral, 2005) to networks composed of several layers (see also Yu et al., 2017). A potential benefit of using this MPC is that it does not depend on interlayer edge weights, which thus do not need to be calculated. As shown in Guillon et al. (2017), using an MPC yields better classification accuracy and sensitivity than using only monolayer network diagnostics.

In this paper, we investigate how to translate the dynamics of different brain regions into a frequency-based (i.e., functional) multilayer network, in which individual layers account for coordination within a given frequency band. We focus specifically on the consequences of analyzing a multiplex network versus a more general multilayer one. The former allows interlayer connections only between the same brain region (i.e., node-layer) in different network layers, so coupling between oscillations in different frequency bands occurs only between the same brain region, whereas the latter allows one to model coordination between any brain region at any frequency band. We use resting-state MEG recordings because of their high temporal resolution (on the order of milliseconds), which makes it possible to analyze a broad spectrum of frequency bands (de Pasquale et al., 2010; van Diessen et al., 2015). In our case, a set of MEG signals consist of *N* time series, each of which comes from a sensor that captures the activity above a different cortical region. We then filter signals at four frequency bands (theta, alpha, beta, and gamma) and construct, for each individual, a four-layer functional multilayer network from the dynamical coordination within and between frequency bands. The existence of interlayer edges in frequency-based functional networks relies on the phenomenon of cross-frequency coupling (CFC), which is responsible for integrating brain activity at different spatial and temporal scales (Canolty et al., 2006). The quantification of CFC is a hard task, because the interplay between frequency bands is very intricate (Aru et al., 2015). To address this issue, one can set the weights of the interlayer edges to a value *p*, which one can estimate by using an optimization procedure. For example, it is possible to construct multiplex networks of two groups of individuals with different profiles and determine a value of *p* that best distinguishes between the two groups (see, e.g., De Domenico, Sasai, & Arenas, 2016). However, we adopt a different strategy: We obtain the weights of the interlayer edges directly from time series by calculating mutual information (MI; MacKay, 2003). We thereby capture heterogeneity (in the weights) of both intralayer edges and interlayer edges, and we investigate the influence of such heterogeneity on the spectral properties of frequency-based functional networks.

Using both synthetic network models and data from laboratory experiments, we investigate the effects that heterogeneity of interlayer edge weights have on the spectral properties of both multiplex and more general multilayer networks. Specifically, we focus on the algebraic connectivity *λ*_2_, which is closely related to both structural and dynamical properties of networks (Masuda, Porter, & Lambiotte, 2017; Newman, 2010; Van Mieghem, 2011). On one hand, algebraic connectivity is an indicator of modular structure in networks (Fortunato & Hric, 2016). In the framework of multilayer networks, one can interpret the value of *λ*_2_ and how it changes as a function of interlayer coupling strength as a way to quantify structural integration and segregation of different network layers (Radicchi & Arenas, 2013); also see the discussions in DeFord & Pauls (2017). On the other hand, 1/*λ*_2_ is proportional to the time required to reach equilibrium in a linear diffusion process (Gómez et al., 2013). Additionally, the time *t*_sync_ to reach synchronization of an ensemble of phase oscillators that are linearly and diffusively coupled is also proportional to 1/*λ*_2_, and it is known that *t*_sync_ and 1/*λ*_2_ are positively correlated in some situations with nonlinear coupling (Almendral & Díaz-Guilera, 2007). A recent investigation of the consequences of modifying interlayer edge weights in multiplex networks has illustrated that enhancing interlayer coupling tends to shorten the time to achieve interlayer synchronization in a Kuramoto model of coupled oscillators (Allen-Perkins, Albuquerque de Assis, Pastor, & Andrade, 2017).

In the framework of functional brain networks, algebraic connectivity has been used as an indicator of AD, such as in de Haan et al. (2012), who obtained statistically significant differences for *λ*_2_ of functional networks obtained from MEG in a comparison of a group of patients suffering from AD with a control group. Phillips, McGlaughlin, Ruth, Jager, and Soldan (2015) calculated the value of *λ*_2_ in a group of individuals with mild cognitive impairment and AD, although they did not report significant differences between them. Computing *λ*_2_ is also necessary for calculating the most standard type of synchronizability parameter, which is the ratio *λ*_*N*_/*λ*_2_, where *λ*_*N*_ is the largest eigenvalue of the combinatorial Laplacian matrix (Boccaletti, Latora, Moreno, Chavez, & Hwang, 2006). In a series of studies, the synchronizability of brain networks was calculated for different frequency bands (Bassett, Meyer-Lindenberg, Achard, Duke, & Bullmore, 2006), during epileptic seizures (Schindler, Bialonski, Horstmann, Elger, & Lehnertz, 2008), and for schizophrenic individuals (Siebenhühner, Weiss, Coppola, Weinberger, & Bassett, 2013). We aim to improve the interpretation of algebraic connectivity for functional brain networks, and we thus investigate (i) how the fact that a considerable fraction of all possible interlayer edges are not present in multiplex networks leads to a deviation from the theoretical values expected for *λ*_2_ and (ii) how these deviations are related to the mean weight of the interlayer edges. We thereby scrutinize the consequences of using a multiplex formalism, in which only CFC inside the same brain region is allowed, instead of employing a fully multilayer approach (i.e., without any restrictions on the type of coupling that one considers).

## RESULTS

### Constructing Frequency-Based Multilayer Networks

In Figure 1, we illustrate the process of constructing frequency-based multiplex and multilayer brain networks. Our starting point is a data set of MEG recordings of a group of *q* = 89 individuals during resting state (see Materials and Methods for details), but one can use other experimental paradigms—including different brain-imaging techniques, such as electroencephalography (EEG) or fMRI—to construct multilayer networks with the same procedure. Specifically, we record MEG activity at *N* cortical regions, with 235 ≤ *N* ≤ 246 (see the Supplementary Information for details; Buldú & Porter, 2017), and we then clean the data to remove artifacts and obtain corresponding unfiltered signals. We thereby analyze the signal recorded by each sensor instead of carrying out a source reconstruction. We then band-pass filter each signal to obtain four different filtered time series for each brain region. We use the four classical frequency bands: theta [3–8] Hz, alpha [8–12] Hz, beta [12–30] Hz, and gamma [30–100] Hz. The number *l* of layers of the multilayer network is the number of different frequencies that we examine (and 2 ≤ *l* ≤ 4 in our work), and the *N* nodes in each layer are associated with the dynamics of the *N* sensors filtered at a given frequency band. We number the nodes so that nodes *n*, *n* + *N*, *n* + 2*N*, …, *n* + *lN* (with *n* ∈ {1, … *N*}) correspond to the signals of the same brain region *n* at the *l* different frequency bands (i.e., layers).

We then quantify coordination between each pair of nodes of a multilayer network, regardless of which layers they are in, using mutual information (see Materials and Methods). Calculating mutual information (MI) between time series of the same frequency band yields intralayer connections between brain regions (see edge type “1” in the bottom-left plot of Figure 1A for an example), so each layer corresponds to a specific frequency band. Edges between the signals of the same sensor at different frequency bands result in interlayer connections between layers (see edge type “2”). Such “diagonal” interlayer edges are the only type of interlayer edges that are allowed in multiplex networks (Kivelä et al., 2014). Finally, cross-frequency coupling between different brain regions yields the other (“nondiagonal”) interlayer edges in a full multilayer network (see edge type “3”). As we show in Figure 1B, we thereby obtain a supra-adjacency matrix, where blocks along the diagonal account for intralayer connections (layers alpha and beta in the two-layer example) and blocks off of the diagonal, marked as **P**^*αβ*^ and **P**^*βα*^, encode the interlayer edges. Because MI_*ij*_ = MI_*ji*_ (see Equation 4 in Materials and Methods) for time series of nodes *i* and *j*, the supra-adjacency matrix is symmetric, so **P**^*αβ*^ = [**P**^*βα*^]^*T*^.

Importantly, although we have chosen to use MI, there are a diversity of similarity measures for capturing amplitude–amplitude and phase–amplitude correlations between different frequency bands (see Aru et al., 2015 for a review of cross-frequency coupling measures), and each measure has its own advantages and drawbacks. Nevertheless, as we will see, the same methodological implications exist no matter which specific measure one uses to evaluate coordination between brain sites.

In our discussion, we focus on the analysis of a two-layer network with alpha and beta layers. We will discuss the consequences of considering alternative frequency bands and numbers of layers in the last section of the paper (see Conclusions and Discussion).

Our starting point is to compare the results from four different constructions of functional networks:

1.  *Unfiltered functional networks*. We obtain these networks from the original (unfiltered) signals of each brain region—that is, without decomposing the signals into different frequency bands—so these are monolayer networks.
2.  *Aggregated networks*. We obtain these networks from componentwise addition of the weights of the alpha and beta layers to form monolayer networks. (Note that this is a uniform aggregation.)
3.  *Multiplex networks*. Each layer corresponds to a specific frequency band (as explained above), and interlayer edges are allowed only between node-layers that correspond to the same brain region.
4.  *Full multilayer networks*. These include the same layers as their multiplex counterparts, but they allow all possible interlayer edges.

In Figure 2, we show the probability density functions (PDFs) for a group of *q* = 89 individuals of the values of *λ*_2_, the standard deviation of the interlayer edge weights (to quantify their heterogeneity), and the percentage of missing interlayer edges (also see Table 1).

**Table 1.** For each kind of network (see the first column), we show the mean standard deviation $\overset{-}{\sigma }$ of the weights of the edges (unfiltered and aggregated) and interlayer edges (for both multiplex networks and full multilayer networks) and the corresponding percentage of missing edges. (See the Supplementary Information for more details; Buldú & Porter, 2017.)

Network	$\overset{-}{\sigma }$	Missing Edges
Unfiltered	0.0823	22.75%
Aggregated	0.1567	4.42%
Multiplex	0.0618	52.17%
(Full) Multilayer	0.0611	44.01%

In column one of Figure 2, we observe that the (monolayer) unfiltered functional network has a similar mean and standard deviation of *λ*_2_ as the multilayer network (also see Table 1). However, the aggregated networks tend to have larger values of *λ*_2_, which makes sense, as we construct an aggregated network by adding the weights of the two layers (alpha and beta), and the total “strength” of the network (i.e., the sum of all of its edge weights) is close to double the strength of each layer. Importantly, the mean *λ*_2_ for the multiplex networks is two orders of magnitude smaller than the mean *λ*_2_ of the multilayer networks. We expect this discrepancy, because we construct a multiplex network by deleting all interlayer edges of a full multilayer network, except for edges (so-called “diagonal” edges) that link the same “physical” nodes (i.e., the same brain regions). Thus, the total strength of the interlayer matrix **P**^*αβ*^ is considerably smaller in multiplex networks than in corresponding full multilayer networks. Because *λ*_2_ is an indicator of the amount of interconnections between communities in a network (Newman, 2010), one expects such drastic edge removals to yield a smaller value of *λ*_2_, as one can construe layers as communities with a small number of edges between them (only *N* of the *N*^2^ possible interlayer edges of a full multilayer network).

In columns two and three of Figure 2, we quantify the heterogeneity and the number of missing interlayer edges of the four different functional networks. In column two, we plot the PDFs of the standard deviation of all edges (for the unfiltered and aggregated networks) and interlayer edges (for the multiplex and multilayer networks). In all cases, we observe that the functional networks that we construct from experimental data have non-negligible heterogeneity (also see Table 1). Again, aggregated networks have values that are roughly twice as large as those of the other kinds of networks because of the (rough) doubling of the mean strength. In column three, we show that there is a large percentage of missing edges. This arises from the fact that we construe edges whose weights are smaller than those obtained with an appropriate surrogate time series as not being statistically significant, so we set their values to 0 (see Materials and Methods for details). The (monolayer) aggregated networks have the smallest mean percentage, followed by the (monolayer) unfiltered networks, and then the two types of multilayer networks. For the multiplex and full multilayer networks, the percentage of missing edges, which is larger than 40% in both cases, refers to the number of all possible interlayer edges. Note that missing edges are unavoidable in functional brain networks, because not all brain regions communicate with each other through direct connections^2^. Moreover, the different amounts of coordination between pairs of brain regions lead to functional networks with heterogeneous weights.

### Heterogeneity and Missing Interlayer Edges in Multiplex Networks

Given our prior observations, a crucial question arises: What are the consequences of heterogenous and missing interlayer edges, both of which are intrinsic features of brain-imaging data, on multiplex and full multilayer functional networks? More specifically, how do they affect the value of *λ*_2_, and what are the ensuing structural and dynamical implications of these differences?

With the aim of answering the above questions, we perform a series of numerical computations in which we compare the theoretical values of *λ*_2_ in multiplex and full multilayer networks from homogeneous interlayer-edge distributions with ones from networks with heterogeneous and missing interlayer edges.

We start with a two-layer multiplex network, whose layers alpha and beta have *N*_*α*_ = *N*_*β*_ nodes and *L*_*α*_ = *L*_*β*_ intralayer edges, respectively. We number the nodes of the alpha layer from *k* = 1 to *k* = *N*_*α*_ and the nodes of the beta layer from *m* = *N*_*α*_ + 1 to *m* = *N*_*αβ*_ = *N*_*α*_ + *N*_*β*_. The matrices **M**^*α*^ and **M**^*β*^ are the corresponding adjacency matrices for each layer. We then introduce *l*_*c*_ connector edges (i.e., diagonal interlayer edges) between each node *k* of the alpha layer to its corresponding node *m* = *k* + *N*_*α*_ of the beta layer to construct a multiplex network. We suppose that intralayer edges have weight *w*_*i*′*j*′_^intra^ = 1 (i.e., for *i*′ and *j*′ both belonging either to the alpha layer or to the beta layer), and we set the weights of the interlayer edges to *w*_*km*_^inter^ = *p*_*km*_ (i.e., for *k* ∈ *α* and *m* ∈ *β*), where *p*_*km*_ are the elements of a vector $\vec{p}$ of the weights of the interlayer connections. Under these conditions, we obtain a supra-adjacency matrix **M**^*αβ*^ that consists of two diagonal blocks (**M**^*α*^ and **M**^*β*^) and two off-diagonal blocks (**P**^*αβ*^ and **P**^*βα*^, where $P^{\alpha \beta }={\left[P^{\beta \alpha }\right]}^{T}=\vec{p}\;I$, where $\vec{p}$ is a row vector). That is,

$$M^{\alpha \beta }=\left(\begin{array}{cc} M^{\alpha } & \vec{p}\;I\\ \vec{p}\;I & M^{\beta } \end{array}\right),$$ (1)

where I is an identity matrix. Following the same procedure, one can also extend the definition of a supra-adjacency matrix to include an arbitrary number of layers. For example, if one considers layers for each of the theta, alpha, beta, and gamma bands (see Materials and Methods), one obtains a supra-adjacency matrix **M**^*θαβγ*^.

The combinatorial supra-Laplacian matrix L^*αβ*^ of the multiplex network is

$$L^{\alpha \beta }=\left(\begin{array}{cc} L^{\alpha }+\vec{p}\;I & -\vec{p}\;I\\ -\vec{p}\;I & L^{\beta }+\vec{p}\;I \end{array}\right),$$ (2)

where the layer combinatorial Laplacians L^*α*,*β*^ have coordinates

$$L_{ij}^{\alpha ,\beta }=\left\{\begin{array}{cc} s_{i}\;, & \;\text{if}\;i=j\\ -1\;, & \;\text{if}\;i\;\text{and}\;j\;\text{are adjacent}\\ 0\;, & \;\text{otherwise} \end{array}\;,\right.$$ (3)

and $s_{i}=\sum_{i\neq j}w_{ij}$ is the weighted degree (i.e., total weight of incident edges) of node *i*.

**Figure 3.**
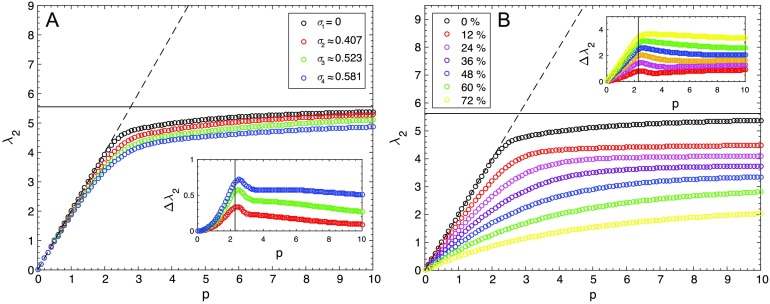
Consequences of heterogenous and missing interlayer edges in a multiplex network. Algebraic connectivity *λ*_2_ of the combinatorial supra-Laplacian matrix L^*αβ*^. Each of the two layers is a *G*(*N*, *p*_con_) Erdős–Rényi network with *N* = 250 nodes and a connection probability of *p*_con_ = 0.25. Each circle corresponds to a mean over 100 realizations. **(A)** We quantify the heterogeneity of the interlayer edges with the standard deviation *σ* of their weights: (i) *σ* = 0 (black circles), (ii) *σ* ≈ 0.407 (red circles), (iii) *σ* ≈ 0.523 (green circles), and (iv) *σ* ≈ 0.581 (blue circles). Lines correspond to analytical solutions for the case *σ* = 0 (i.e., a homogenous multiplex network). The dashed line is *λ*_2_ = 2*p*, and the solid line is $\lambda_{2,\text{agg}}=\frac{1}{2}\lambda_{2}(L^{\alpha }+L^{\beta })$ (Radicchi & Arenas, 2013; Sahneh et al., 2015). In the inset, we show Δ*λ*_2_, the difference of *λ*_2_ between the homogeneous multiplex network and a multiplex network with heterogeneous interlayer edges. (In the inset, the solid vertical line indicates the mean value 〈p*〉 of the transition point.) **(B)** Algebraic connectivity *λ*_2_ of the multiplex networks as a function of the number of missing interlayer edges. In the inset, we show Δ*λ*_2_, the difference of *λ*_2_ between the homogeneous multiplex network (i.e., *σ* = 0 and all possible interlayer edges) and the multiplex networks with missing interlayer edges. As before, the solid vertical line indicates the mean value 〈p*〉 of the transition point.

In Figure 3, we show the consequences of heterogeneity in the distribution of the interlayer-edge weights of the multiplex network **M**^*αβ*^. In this example, the multiplex network has an interlayer connectivity matrix $P^{\alpha \beta }=\vec{p}\;I$, where I is an *N*_*α*_ × *N*_*α*_ (equivalently, *N*_*β*_ × *N*_*β*_, as *N*_*α*_ = *N*_*β*_ in this example) identity matrix and $\vec{p}=p\vec{h}$ is a row vector that controls the weights of interlayer edges, where *p* modulates the vector’s magnitude and the vector $\vec{h}$ encodes the heterogeneity of the interlayer edges. We set the elements of $\vec{h}$ to follow a uniform distribution over the interval [*h*_min_, *h*_max_]. These elements have a mean value of $\overset{-}{h}=1$ and a standard deviation of *σ*. We set $\overset{-}{h}=1$ and construct networks with interlayer-edge-weight heterogeneities that range from *σ* = 0 (blue circles) to *σ* ≈ 0.581 (green circles). We then examine the interplay between the weights of the interlayer edges and the heterogeneity by increasing the value of *p*. Note that *σ* = 0 corresponds to what we call a *homogeneous multiplex network*, which has uniformly-weighted interlayer edges (i.e., $\vec{p}=p$ for all interlayer edges). We obtain the results in Figure 3 by calculating a mean over 100 realizations of two-layer networks with the *G*(*N*, *p*_con_) Erdős–Rényi (ER) model (with *p*_con_ = 0.25) in each layer and *N* = *N*_*α*_ = *N*_*β*_ = 250 nodes (Newman, 2010).

As explained in Radicchi and Arenas (2013), modifying the weight parameter *p* of the interlayer edges has important consequences for the value of *λ*_2_ for homogeneous multiplex networks. The existence of two regimes of qualitatively distinct dynamics, separated at a transition point p*, was discussed in Radicchi and Arenas (2013) (and in various subsequent papers): When *p* ≪ p*, the algebraic connectivity *λ*_2_ follows the linear relation *λ*_2_ = 2*p*; for *p* ≫ p*, however, the value of *λ*_2_ approximates that of the (uniformly) aggregated network [i.e., $\lambda_{2,\text{agg}}=\frac{1}{2}\lambda_{2}(L^{\alpha }+L^{\beta })$]. In Figure 3, the dashed and solid lines indicate the theoretical predictions of *λ*_2_ in the homogeneous case for small (dashed) and large (solid) values of *p*. We obtain a mean value of p* ≈ 2.870 by locating the intersection between the eigenvalues *λ*_2_ and *λ*_3_ of the supra-Laplacian matrix L^*αβ*^ (Sahneh, Scoglio, & Van Mieghem, 2015).

In Figure 3A, we illustrate that the heterogeneity of the (weights of the) interlayer edges leads to non-negligible differences in *λ*_2_. Furthermore, values of *p* near p* have the maximum discrepancy between heterogeneous cases (colored circles) and the homogeneous case (black circles). In studies of multiplex networks, it is simplest to take interlayer edge weights to be homogeneous (i.e., given by a constant value *p*), especially when it is not clear how to estimate their values Kivelä et al. (2014). When possible, however, it is desirable to be more sophisticated, and it is possible to quantify the coupling strength between different frequency bands in various ways when studying frequency-based functional brain networks (Aru et al., 2015). The choice of incorporating versus ignoring heterogeneity of inter-frequency edge weights then leads to unavoidable differences in the estimation of *λ*_2_, especially near the transition point p*. It is also important to think about the different interlayer edge weights that one obtains from different measurements of coupling strength (see Materials and Methods).

Discrepancies in the values of *λ*_2_ are typically even larger when some interlayer edges are missing. In Figure 3B, we show the effects of removing some percentage of the interlayer edges in a “holey” multiplex network (i.e., a multiplex network with an interlayer connectivity matrix **P**^*αβ*^ whose elements are either 1 or 0 along the diagonal and are 0 in all other entries). We observe that increasing the number of missing interlayer edges causes the multiplex networks to have drastically reduced values of *λ*_2_. When *p* ≪ p*, the value of *λ*_2_ always grows with a slope that is smaller than 2*p*; for *p* ≫ p*, however, the value of *λ*_2_ never reaches the value of *λ*_2_ for the aggregated network. Finally, values of *p* close to p* again have the largest discrepancies with respect to the homogeneous case.

### Mutilplex Networks Versus Full Multilayer Networks

**Figure 4.**
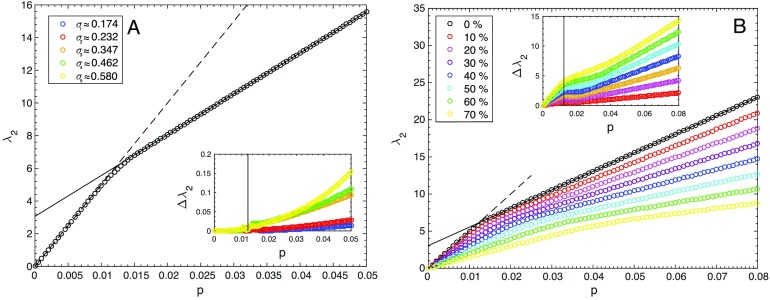
Edge heterogeneity and missing interlayer edges in a full multilayer network. Algebraic connectivity *λ*_2_ of the combinatorial supra-Laplacian matrix L_*αβ*_ (black circles) as a function of the weight parameter *p* of the interlayer edges. Each of the two layers is a *G*(*N*, *p*_con_) Erdős–Rényi network with *N* = 250 nodes and a connection probability of *p*_con_ = 0.25. Each circle corresponds to a mean over 100 realizations of such multilayer networks. **(A)** Algebraic connectivity *λ*_2_ for a homogeneous interlayer connectivity matrix **P**^*αβ*^ = *p***C**, where each of the elements of **C** is *c*_*ij*_ = 1. The dashed line, given by *λ*_2_ = 2*p*〈*c*〉, corresponds to the analytical solution for a homogeneous matrix **P**^*αβ*^, where **C** has a mean weighted degree of 〈*c*〉 = 250. The solid line is given by *λ*_2_ = *λ*_2,min(*α*,*β*)_ + *λ*_2_(*p***C**), where *λ*_2,min(*α*,*β*)_ is the value of the smaller *λ*_2_ of the two isolated layers. The inset shows the differences Δ*λ*_2_ between a multilayer network with homogeneous interlayer connectivity matrix **P**^*αβ*^ and a series of multilayer networks with increasing heterogeneity (quantified by the standard deviation *σ*) in **P**^*αβ*^. Differences with the homogeneous case increase after the mean 〈p*〉 of the transition point (vertical solid line). **(B)** Algebraic connectivity *λ*_2_ of the full multilayer networks as a function of the percentage of missing interlayer edges. (See the values of the percentages in the legend.) In the inset, we show the increment of *λ*_2_ versus the number of missing interlayer edges.

We now identify the qualitative differences (and some of their consequences) between multiplex networks and full multilayer networks. The latter have an interlayer connectivity matrix **P**^*αβ*^ = *p***C**, where **C** has elements *c*_*ij*_ that account for the weight between each pair {*i*, *j*} of nodes *i* ∈ *α* and *j* ∈ *β*, and the parameter *p* allows one to modulate the mean weight of the interlayer edges. In Figure 4A, we connect the same layers as in our previous example, but now we use a homogeneous interlayer connectivity matrix **P**^*αβ*^ with weights *p*_*ij*_ = *p* (i.e., *c*_*ij*_ = 1 for all *i* and *j*). Increasing *p* from 0 leads to the transition point p* ≈ 0.013, which we obtain from the intersection of the eigenvalues *λ*_2_ and *λ*_3_ of the combinatorial supra-Laplacian L^*αβ*^. We observe for *p* < p* that the value of *λ*_2_ follows the linear function *λ*_2_ = 2*p*〈*c*〉 (see the dashed line in Figure 4A), where 〈*c*〉 = 250 is the mean weighted degree of the matrix **C**, as demonstrated in Radicchi and Arenas (2013). Interestingly, after the transition point *p* = p*, one can describe the value of *λ*_2_ by the function *λ*_2_ = *λ*_2,min(*α*,*β*)_ + *λ*_2_(*p***C**), where *λ*_2,min(*α*,*β*)_ is the value of the smaller *λ*_2_ of the two isolated layers. As we show in the inset of Figure 4A, when we introduce a certain amount of heterogeneity into the interlayer connectivity matrix, we observe slight differences from homogeneous case. Specifically, for heterogeneous cases, these differences are larger for values of *p* above the mean 〈p*〉 of the transition point.

As we saw in our analysis of multiplex networks, deleting interlayer edges from a full multilayer network increases the discrepancy in *λ*_2_ values between networks with homogeneous interlayer edges and networks with heterogeneous ones. As we show in Figure 4B, the value of *λ*_2_ decreases with the number of missing edges, which one should expect, because having a smaller number of interlayer edges implies that one needs larger edge weights to maintain the same amount of interlayer coupling. Interestingly, the deviation from the theoretical predictions is significant even for *p* < 〈p*〉. Additionally, for *p* > 〈p*〉, we observe (as expected) a change in the slope of *λ*_2_, but the theoretical predictions given by *λ*_2_ = *λ*_2,min(*α*,*β*)_ + *λ*_2_(*p***C**) (solid line) begin to fail, leading to a discrepancy that increases with the percentage of missing edges.

**Figure 5.**
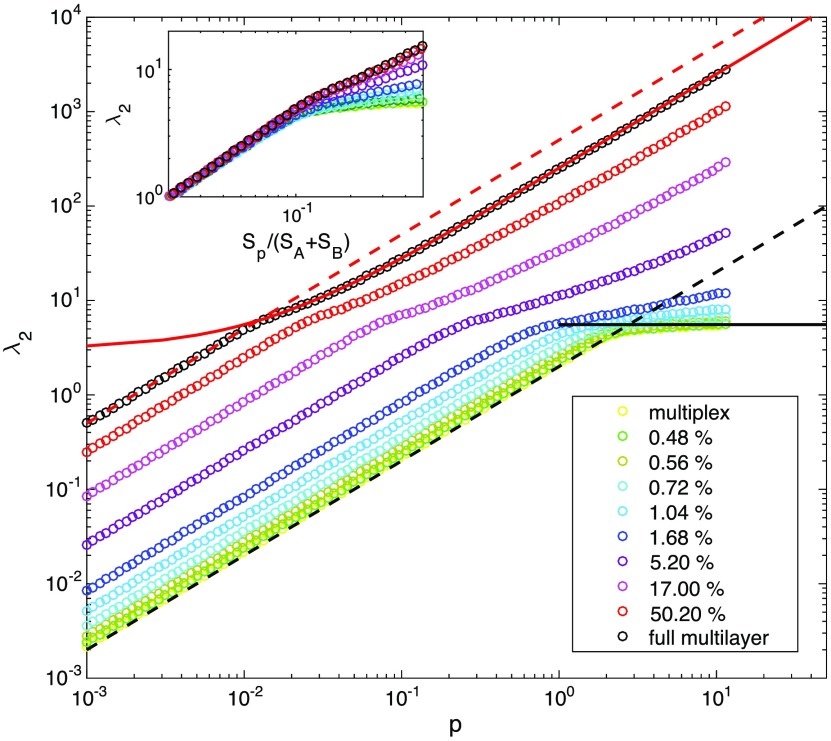
Transition from a multiplex network to a full multilayer network. Algebraic connectivity *λ*_2_ of the combinatorial supra-Laplacian matrix L^*αβ*^ for different values of the percentage of edges in the interlayer connectivity matrix **P**^*αβ*^. Departing from a multiplex network, we add interlayer edges uniformly at random and calculate the percentage of existing edges in **P**^*αβ*^. Each circle corresponds to a mean over 100 realizations. We set all active edges of the interlayer connectivity matrix **P**^*αβ*^ to *p*_*ij*_ = *p*. The dashed lines correspond to the analytical solutions for the full multilayer network (black dashed line; *λ*_2_ = 2*p*〈*c*〉, with 〈*c*〉 = 250) and the multiplex network (red dashed line; *λ*_2_ = 2*p*) for *p* < 〈p*〉. The solid curves are the theoretical solutions when *p* > 〈p*〉 for the full multilayer network (black line; *λ*_2_ = *λ*_2,min(*α*,*β*)_ + *λ*_2_(*p***C**)) and the multiplex network (red curve; $\lambda_{2,\text{agg}}=\frac{1}{2}\lambda_{2}(L^{\alpha }+L^{\beta }))$. In the inset, we show *λ*_2_ versus *S*_*P*_/(*S*_*α*_ + *S*_*β*_), where *S*_*P*_, *S*_*α*_, and *S*_*β*_ are, respectively, the strength of the interlayer connectivity matrix **P**^*αβ*^, the strength of the alpha layer, and the strength of the beta layer. Note that differences between the values of *λ*_2_ of the multilayer structures increase significantly for $S_{P}/(S_{\alpha }+S_{\beta })⪆0.1$.

Because full multilayer networks have up to *N*^2^ interlayer edges, whereas multiplex networks can have only *N* of them, the former tend to have interlayer connectivity matrices with larger strengths $S_{P}=\sum_{ij}p_{ij}$. In Figure 5, we show the algebraic connectivity *λ*_2_ of the combinatorial supra-Laplacian matrices L^*αβ*^ for a series of networks with identical layers and interlayer strength *S*_*P*_, but with a different number of interlayer edges, ranging from a multiplex network (with *N* interlayer edges) to a full multilayer network with no nonzero entries (i.e., with *N*^2^ interlayer edges). The dashed lines correspond to the analytical solutions for the full multilayer network (black dashed line; *λ*_2_ = 2*p*〈*c*〉, with 〈*c*〉 = 250) and the multiplex network (red dashed line; *λ*_2_ = 2*p*) when *p* < p*. The solid curves are the corresponding theoretical solutions when *p* > 〈p*〉 for the full multilayer network (black line; *λ*_2_ = *λ*_2,min(*α*,*β*)_ + *λ*_2_(*p***C**)) and the multiplex network (red curve; $\lambda_{2,\text{agg}}=\frac{1}{2}\lambda_{2}(L^{\alpha }+L^{\beta }))$. We observe the effect that adding interlayer edges to multiplex networks has on the value of *λ*_2_ and the associated transition from a multiplex network to a full multilayer architecture. Interestingly, the different numbers of interlayer edges in the two types of networks leads to a difference in the position of 〈p*〉 (which one can infer by looking at the change of slope of *λ*_2_) that can reach several orders of magnitude.

The inset of Figure 5 illustrates the same results normalized by the total strength of the interlayer connectivity matrices. That is, we show *S*_*P*_/(*S*_*α*_ + *S*_*β*_), where $S_{\alpha }=\sum M_{ij}^{\alpha }$ and $S_{\beta }=\sum M_{ij}^{\beta }$, respectively, are the strengths of layers alpha and beta. This allows us to compare networks with the same value of *S*_*P*_, regardless of whether they are close to a multiplex architecture or to a fully multilayer architecture with no nonzero entries. For *p* < 〈p*〉, we obtain similar values of *λ*_2_ for all network architectures. It is only for *p* > 〈p*〉 that the particular structure of the interlayer connectivity matrix begins to play a role in the value of *λ*_2_. We observe that differences start to arise at *S*_*P*_/(*S*_*α*_ + *S*_*β*_) ≈ 0.1, which is a relatively small value.

Although we used ER intralayer networks in our above calculations, we obtain similar results for other network models. In particular, the results are qualitatively the same when we construct the intralayer networks using a Barabási–Albert (BA) model (Barabási & Albert, 1999). See the Supplementary Information for details (Buldú & Porter, 2017).

### The Meaning of *λ*_2_ in Experimental Data

Now that we have analyzed the effects of edge-weight heterogeneity and the number of missing interlayer edges, let’s revisit the multiplex and multilayer networks that we constructed from the MEG recordings. As we saw in Figure 2, both edge-weight heterogeneity and missing interlayer edges occur in our experimental data, and it is thus desirable to investigate how close the experimental networks that we are analyzing are to a transition point p* and how this proximity (or lack thereof) influences the expected value of *λ*_2_.

**Figure 6.**
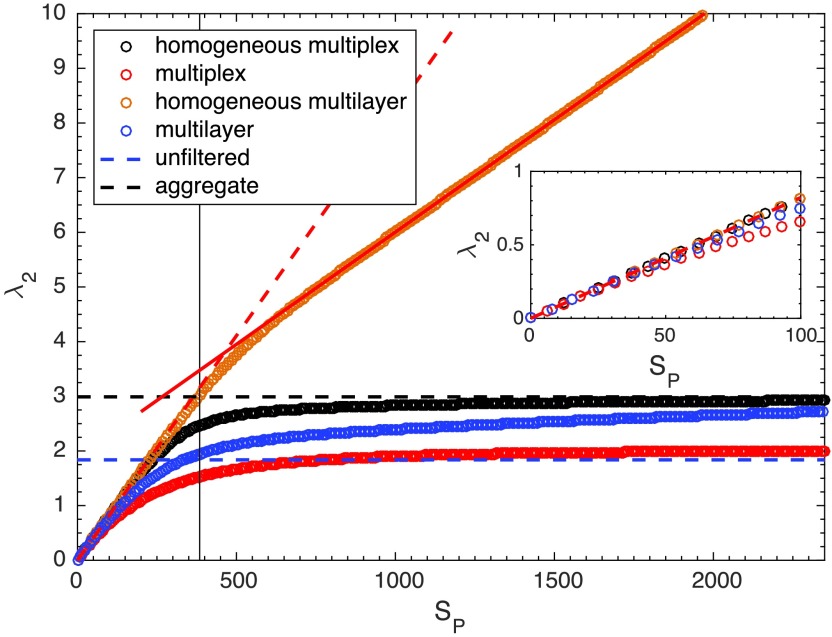
Edge heterogeneity and missing interlayer edges in frequency-based functional brain networks. Algebraic connectivity *λ*_2_ of the combinatorial supra-Laplacian matrix L^*αβ*^ as a function of the mean strength of the interlayer connectivity matrix **P**^*αβ*^ = *p***C**, where **C** encodes the weights of the interlayer edges, for four different types of networks: (i) homogeneous multiplex networks (black), (ii) heterogeneous multiplex networks (red), (iii) homogeneous full multilayer networks (orange), and (iv) heterogeneous full multilayer networks (blue). Each circle is a mean over 89 individuals. The blue and black dashed lines, respectively, are the values of *λ*_2_ for the unfiltered and aggregated networks. The red dashed line corresponds to *λ*_2_ = 2*p*〈*c*〉, where 〈*c*〉 is the mean of the weighted degree. The red solid line is given by *λ*_2_ = *λ*_2,min(*α*,*β*)_ + *λ*_2_(*p***C**), where *λ*_2,min(*α*,*β*)_ is the value of the smaller *λ*_2_ of the two isolated layers.

In Figure 6, we show the values of *λ*_2_ that we obtain for four different network reconstructions based on the MEG data: a homogeneous multiplex network (black circles), a heterogeneous multiplex network (red circles), a homogeneous full multilayer network (blue circles), and a heterogeneous full multilayer network (cyan circles). Each network has two layers—one for the alpha band and one for the beta band—and each point corresponds to the mean over the group of 89 individuals. We obtain the heterogeneous versions of the multiplex and multilayer networks by calculating MI values between the brain regions in the frequency bands, as we described previously (also see Materials and Methods). Second, we construct the homogeneous versions of both the multiplex and multilayer networks by assigning the same weight 〈*c*〉 to all interlayer edges, where 〈*c*〉 is the mean of the weights of the interlayer edges in their heterogeneous counterparts. Note that, in this case, the homogeneous multiplex and multilayer networks do not correspond to real functional networks; instead, they are reference networks that we use to quantify the consequences on *λ*_2_ of the intrinsic edge heterogeneity and missing edges in real functional networks.

To assess how close the real networks are to the transition point p*, we multiply the values of the interlayer edges by a parameter *p*, which we increase from *p* = 0 to a value above 1. We then calculate the strength *S*_*P*_ of the corresponding interlayer connectivity matrix **P**^*αβ*^ to allow a comparison between the multiplex network and the multilayer networks, and we plot the value of *λ*_2_ versus *S*_*P*_. The red dashed line in Figure 6 corresponds to the theoretical predictions for *p* < p* (i.e., *λ*_2_ = 2*p*〈*c*〉, where 〈*c*〉 is the mean weighted degree of the nodes in the interlayer connectivity matrix **P**^*αβ*^). The black and blue dashed lines are, respectively, the value of *λ*_2_ for the aggregated network divided by 2 (i.e., $\lambda_{2,\text{agg}}=\frac{1}{2}\lambda_{2}(L^{\alpha }+L^{\beta }))$ and the value of *λ*_2_ for the unfiltered (monolayer) functional network. The vertical solid line corresponds to the case *p* = 1 for the mean of the heterogeneous full multilayers networks (i.e., the networks that we obtain by taking into account all interlayer correlations without modifying their weights). Interestingly, this network occupies the region in which the evolution of *λ*_2_ with respect to *p* changes slope from *λ*_2_ = 2*p*〈*c*〉 to *λ*_2_ = *λ*_2,min(*α*,*β*)_ + *λ*_2_(*p***C**), which suggests that the frequency-based multilayer networks that we are analyzing are close to the transition point p*. As we have seen, it is near this point where the value of *λ*_2_ is most influenced by the effects of edge heterogeneity and missing interlayer edges. It is also worth noting that the four network representations have rather different values of algebraic connectivity (and hence, we expect, have different spectral properties more generally), except when *p* ≪ p* (see the inset of Figure 6). Although it looks from the plot that the algebraic connectivities of the heterogeneous multilayer networks (cyan circles) and those of the homogeneous multiplex networks (black circles) may converge to the same value for large *S*_*P*_, they eventually cross each other when we further increase *S*_*P*_ (not shown).

**Figure 7.**
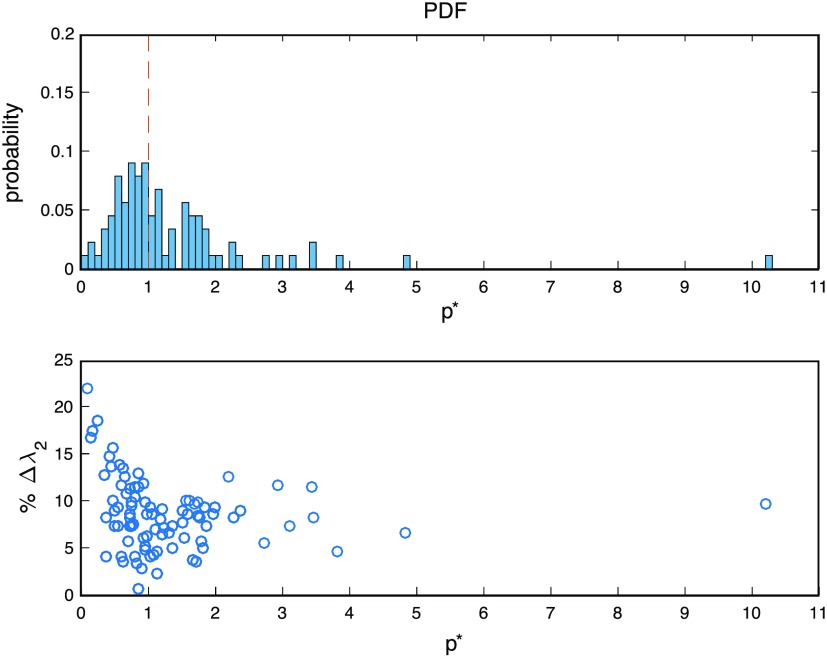
MEG of full multilayer networks and the transition point p*. (Top) PDF of the transition point p* for two-layer networks (of the alpha and beta layers) of the 89 individuals. The dashed line corresponds to p* = 1. (Bottom) Percentage of deviation of *λ*_2_ with respect to the value of the aggregated network: %Δ*λ*_2_ = 100(*λ*_2,agg_ − *λ*_2_)/*λ*_2,agg_, where *λ*_2_ denotes the algebraic connectivity of the full multilayer network. Each circle represents one of the 89 individuals.

In Figure 7, we show (top panel) the PDF of the values of p* for the multilayer networks of each of the 89 individuals and (bottom panel) the percentage of deviation of *λ*_2_ with respect to the value *λ*_2,agg_ of the aggregated network. We observe that the peak of the PDF for p* is near *p* = 1. That is, the multilayer networks that we constructed from empirical data are close to the transition point. Figure 7 also illustrates discrepancies in the values of *λ*_2_ between the aggregated and multilayer networks, indicating that it is necessary to differentiate between the two cases when interpreting the value of *λ*_2_, especially when comparing results from different studies.

**Figure 8.**
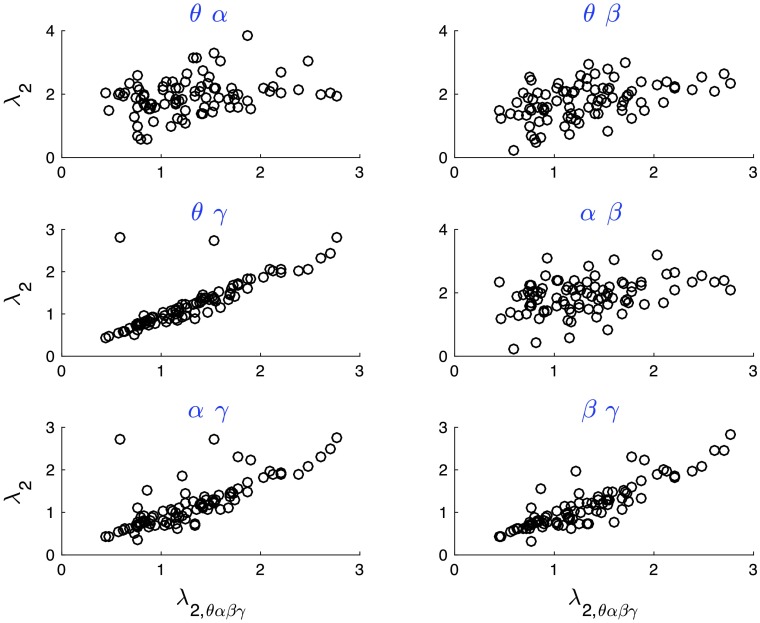
Combining different frequency bands into multilayer networks. We show the algebraic connectivity *λ*_2_ of all possible combinations of two-layer networks versus *λ*_2,*θαβγ*_ of the four-layer full multilayer networks. We construct the layers from the theta (*θ*), alpha (*α*), beta (*β*), and gamma (*γ*) frequency bands. The labels at the top of each plot correspond to the associated frequency bands. Each circle corresponds to one of the 89 individuals.

Finally, we investigate how the combination of layers from different frequency bands affects the value of *λ*_2_. In our analysis thus far, we have focused on a two-layer network that we constructed using the alpha and beta frequency bands, because they are often associated with brain activity during the resting state. Nevertheless, because the signal has been filtered into four different frequency bands, there are eight possible combinations of two layers. In Figure 8, we show the relation between *λ*_2_ for all possible combinations of two-layer networks versus that for the full four-layer multilayer network. Observe the strong correlation of the two-layer networks that include the gamma layer (especially the one that consists of the theta and gamma layers) with the full multilayer network. One can explain such a correlation by inspecting the total strength of each frequency band. In Table 2, we separate the intralayer and interlayer strengths to facilitate interpretation of the results. We observe that the gamma band is the least-active layer, as it is the one with the smallest intralayer strength. Nevertheless, it has the largest interlayer strength (i.e., the sum of the weights of its interlayer edges with all other layers is the largest), so it is the layer that appears to interact most strongly with the other layers. Because (as we have seen) the full multilayer functional networks are close to the mean 〈p*〉 of the transition point, the weight of the intralayer connections has a strong influence on the value of *λ*_2_. Therefore, the two-layer theta–gamma network, which includes the layers with the largest interlayer strengths, is the one with the strongest correlation of *λ*_2_ with the full multilayer network (see Figure 8C).

**Table 2.** Percentage of strength of each layer in a four-layer multilayer network from MEG data. The first column indicates the layer. The second column indicates the percentage of strength of all intralayer edges in the full four-layer multilayer network that come from that layer; that is, for a layer *l* ∈ {*θ*, *α*, *β*, *γ*}, the percentage is given by $100\times \frac{∥M^{l}{∥}_{1}}{∥M^{\theta \alpha \beta \gamma }{∥}_{1}}$, where the operator ∥**M**∥_1_ is the entrywise 1-norm of **M**, corresponding to the sum of all elements of the matrix. The third column gives the percentage of the strength of the interlayer edges. That is, for a layer *l*′, the percentage is $100\times \frac{2\sum_{l\neq l^{\prime }}∥P^{l\prime l}{∥}_{1}}{∥M^{\theta \alpha \beta \gamma }{∥}_{1}}$, where *l*′ ∈ {*θ*, *α*, *β*, *γ*}. Each percentage in the table is a mean over the 89 individuals.

Layer	Intralayer Strength	Interlayer Strength
theta (*θ*)	12.69%	9.53%
alpha (*α*)	31.43%	7.49%
beta (*β*)	12.18%	9.34%
gamma (*γ*)	6.85%	10.47%

## CONCLUSIONS AND DISCUSSION

Using network analysis as a tool for analyzing brain-imaging data, and (more specifically) implementing and studying a multilayer description of brain activity, has both advantages and drawbacks that must be investigated carefully. As we have discussed, it is possible to encode such imaging data either as a multiplex network or as a more general type of multilayer network, but different choices lead to different results, which one must then interpret from a neuroscientific perspective. In our paper, we have analyzed the implications of such a choice on spectral information—and specifically on the algebraic connectivity *λ*_2_, which has been related to structural, diffusion, and synchronization properties of networks (Almendral & Díaz-Guilera, 2007; Gómez et al., 2013; Masuda et al., 2017; Newman, 2010; Radicchi & Arenas, 2013; Van Mieghem, 2011). We have seen how heterogeneity (of the weights) of the interlayer edges of multiplex networks leads to deviations of the theoretical predictions that one obtains when all interlayer edges have an equal weight *p*, and we observed that the deviation is even larger when interlayer edges are missing. The importance of these results, which entail large differences in qualitative dynamics, is underscored by the fact that both edge heterogeneity and missing interlayer edges are common features of brain-imaging data.

It is also important to understand that although a multiplex description of brain networks is an important and useful approach to integrate multivariate information, and it is often an extremely natural approach—such as for integrating anatomical and functional networks, examining the temporal evolution of a functional network, and so on—it is just an initial step towards developing increasingly complete models to better analyze the spatial and temporal complexity of brain networks (Papo, Zanin, & Buldú, 2014). In this quest, a natural step—although certainly not the final step—is to use a full multilayer description for subsequent analyses of frequency-based brain networks.^3^ In this type of network, it is more biologically plausible to represent brain activity as a full multilayer network than as a multiplex network. The reasons are twofold: (i) a brain region does not necessarily coordinate with itself at different frequencies; and (ii) there may also exist CFCs between different brain regions. Consequently, interlayer connections do not necessarily follow the multiplex paradigm, because some of the interlayer edges probably should not be present, whereas others should be included to account for interlayer coupling between different nodes (i.e., different brain regions), thereby necessitating a fully general multilayer approach.^4^ Nevertheless, the use of frequency-based multiplex networks has been an effective strategy in previous work for successfully distinguishing between healthy individuals and individuals who are suffering from schizophrenia (De Domenico, Sasai, and Arenas, 2016), so there are times when a multiplex approach works well.

As we have illustrated with our computations, because the number of interlayer edges scales with *N* in multiplex networks but with *N*^2^ in the multilayer networks that we examined, there are important quantitative differences in the values of the algebraic connectivity in the two types of networks. Nevertheless, the effects of edge-weight heterogeneity and missing interlayer edges on full multilayer networks are qualitatively similar to those in multiplex networks. Specifically, the analytical predictions that we obtained with homogeneous multilayer networks deviate from those of heterogeneous cases, showing an important discrepancy for values of the coupling-strength parameter *p* that are larger than the transition point p*. The effect of missing interlayer edges is particularly dramatic for *p* > p*.

Our analysis of experimental imaging data confirms our results with synthetic networks. The small number of interlayer edges in a multiplex network leaves the network in a region in which layers behave as if they are structurally independent, and a deeper analysis reveals that it is necessary to increase the weights of the interlayer edges from the experimental data by several orders of magnitude to reach the transition point p*. Consequently, using a multiplex representation leads to a network with structurally independent layers, unless one uses extremely large weights (i.e., *p* ≫ 1) for the interlayer edges. However, the weights of the interlayer edges cannot be increased artificially without limits, because eventually they lose their biophysical meaning in attempts to compare them to intralayer edge weights.

Interestingly, our results changed drastically when we used a full multilayer description, in which a brain region whose dynamics are in a given frequency band can now couple with other brain regions in a different frequency band. When we considered all possible interlayer edges, the majority of the 89 individuals in the experiment are close to the transition point p*, even though the percentage of weight in the interlayer edges is always about 10% of that of the intralayer ones (see Table 2). As we showed in our analysis of synthetic data, it is around this point that edge-weight heterogeneity and missing interlayer edges begin to yield important discrepancies from the theoretical results of homogeneous multilayer networks (see Figure 4A).

It is important to note that the percentage of total weight of interlayer edges (relative to the total weight of all edges) depends strongly on how one measures coordination between brain regions. When one uses diagnostics other than mutual information, one calculates different values for the percentages, which can yield multilayer networks either above or below the transition point p*. Thus, no matter what measure one uses, it is mandatory to first calculate the percentage of total edge weight that arises from interlayer edges and to interpret the value of *λ*_2_ with respect to the value of p*.

The fact that, in frequency-based multilayer networks, the weights of interlayer edges strongly influence the value of *λ*_2_ despite being much smaller than the weights of intralayer edges (see Table 2) highlights the importance of adequately evaluating CFCs, which traditionally have been disregarded when representing brain activity in terms of functional networks. As has been discussed prominently in neuroscience, including in critiques of connectomics (Kopell, Gritton, Whittington, & Kramer, 2014), the dynamics matter, and investigations of dynamics must include incorporation and analysis of coupling between different frequency bands. The methodology that is used to quantify interactions between brain regions at different frequencies leads to different values of *λ*_2_; and there are also other important dynamical issues, such as phase–amplitude correlations, that we have not investigated in this paper. (See Bastos & Schoffelen, 2016 for a review of how common reference, volume conduction, field spread, or common input affect the quantification of coordinated activity between brain regions.) These caveats notwithstanding, our analysis illustrates a viable approach for examining the effects of such phenomena on *λ*_2_ (and hence on spectral structure) in multilayer functional networks, and we expect that similar qualitative phenomena will arise in both multiplex and full multilayer networks that are constructed using other choices (e.g., different measures than MI) from the ones that we chose in order to provide a concrete illustration.

We initially studied two-layer networks of the alpha and beta frequency bands, because (i) it is known that these bands incorporate a large portion of the power spectrum of brain activity during resting state and (ii) these frequency bands typically exhibit stronger synchronization between brain regions (e.g., see Brookes et al., 2016) than the others. The alpha and beta bands are thus the most commonly studied frequency bands in resting-state studies, and we followed this tradition. Nevertheless, in our particular case, our comparison between two-layer and four-layer networks illustrates that the gamma band is the one that most influences the spectral properties of the full four-layer multilayer network (see Figure 8). As we showed in Table 2, the theta and gamma layers are the ones whose interlayer edges give the largest contributions, thereby leading to the strongest correlation between a two-layer network (with theta and gamma layers) and the complete (four-layer) multilayer network. This fact highlights the importance of the well-known phase–amplitude correlations between the theta and gamma frequency bands (Aru et al., 2015; Canolty et al., 2006), as the former acts as a carrier of fast-amplitude fluctuations in the latter. Consequently, theta–gamma coupling may be of fundamental importance for understanding the multilayer nature of functional brain networks.

It also worth mentioning that the facts that we are constructing frequency-based multilayer networks from (i) data at the sensor level and (ii) using MI as a measure to quantify mutual interdependency between brain regions leads to unavoidable errors in the quantification of edge weights, due to signal mixing and spurious correlations from common sources. However, to date, there does not exist an error-free methodology to construct functional brain networks, and other alternatives (such as source reconstruction or using different measures to evaluate interdependency between brain regions) have their own drawbacks. For a detailed discussion, see the subsection Methodological Considerations (in Materials and Methods).

Finally, although our calculations with experimental data used resting-state MEG recordings, we expect to observe similar behavior in frequency-based multilayer networks of different origins—whether obtained from any of a large variety of different cognitive or motor tasks, with different brain-imaging techniques, or even if they come from a completely different system (such as functional climate networks; Tsonis & Roebber, 2004). However, despite the generality of our results, it is important to examine richer models of frequency-based brain network, such as ones that include spatial constraints and temporal evolution.

## MATERIALS AND METHODS

### Data Acquisition

The data were made available by the Human Connectome Project (HCP); see http://www.humanconnectome.org/ and Larson-Prior et al. (2013) for details. The experimental data consist of magnetoencephalographic (MEG) recordings of a group of 89 individuals, during resting state, for a period of approximately 2 min. During the scan, subjects were supine and maintained fixation on a projected red crosshair on a dark background. Brain activity was scanned on a whole-head MAGNES 3600 (4D Neuroimaging, San Diego, CA, USA) system housed in a magnetically-shielded room, and it included up to 248 magnetometer channels. The root-mean-squared noise of the magnetometers is about 5 fT/sqrt (Hz) on average in the white-noise range (above 2 Hz). The data were recorded at a sampling rate of *f*_*s*_ ≈ 508.63 Hz. Five current coils attached to a subject, in combination with structural-imaging data and head-surface tracings, were used to localize the brain in geometric relation to the magnetometers and to monitor and partially correct for head movement during MEG acquisition. Artifacts, bad channels, and bad segments were identified and removed from the MEG recordings, which were processed with a pipeline based on independent component analysis to identify and clean environmental and subject artifacts (Larson-Prior et al., 2013). After this process, the number *N* of channels considered for each individual was in the range 235 ≤ *N* ≤ 246 (with a mean of 243.42), because some channels were used as references and others were disregarded.

### Coordination Between Brain Regions

To estimate coordination between brain regions, we first apply a band-pass filter to the preprocessed signals to obtain, for each of the *N* sensors, a set of four different time series, each of which corresponds to a specific frequency band: theta [3–8] Hz, alpha [8–12] Hz, beta [12–30] Hz, and gamma [30–100] Hz. We thereby obtain 4*N* time series of *t* = 149646 points for each of the *q* = 89 individuals. We then order the filtered signals according to their corresponding frequency band, so the time series *X*^*s*^ with *s* ∈ {1, …, *N*} corresponds to the theta band, *X*^*s*^ with *s* ∈ {*N* + 1, …, 2*N*} corresponds to the alpha band, *X*^*s*^ with *s* ∈ {2*N* + 1, …, 3*N*} corresponds to the beta band, and *X*^*s*^ with *s* ∈ {3*N* + 1, …, 4*N*} corresponds to the gamma band. Each of the 4*N* time series corresponds to a node-layer of a frequency-based network. We calculate mutual information (MI) between the time series *X*^*i*^ and *X*^*j*^ of a pair of nodes *i* and *j* with the formula

$${\text{MI}}_{ij}=\underset{u,v}{\sum }p_{uv}\;\;\ln\left(\frac{p_{uv}}{p_{u}p_{v}}\right),$$ (4)

where *p*_*u*_ is the probability that *X*^*i*^ = *x*_*u*_, the quantity *p*_*v*_ is the probability that *X*^*j*^ = *x*_*v*_, and *p*_*uv*_ is the joint probability that *X*^*i*^ = *x*_*u*_ and *X*^*j*^ = *x*_*v*_ at the same time point. We set the number of bins of the PDFs to be $u=v=5\times \lfloor (\sqrt{t/10})\rceil$, where *t* the is the number of time points and ⌊*y*⌉ corresponds to the nearest integer function of the real number *y* (where we round up for .5). When *X*^*i*^ and *X*^*j*^ are independent variables, *p*_*uv*_ = *p*_*u*_*p*_*v*_, and the resulting value of MI_*ij*_ is 0. When *X*^*i*^ = *X*^*j*^ for each time series, MI_*ij*_ achieves its maximum value. Note that MI_*ij*_ = MI_*ji*_, so we obtain an undirected edge between the two time series, and we are disregarding causality. Calculating MI_*ij*_ allows one to detect coordinated activity even for time series that include different frequency bands. See Bastos and Schoffelen (2016) and Pereda, Quiroga, and Bhattacharya (2005) for a review of different measures for quantifying coordination between brain regions and a discussion of their advantages and pitfalls. Note that MI_*i*(*i*+*N*)_ measures the coordination between two different frequency bands in the same brain region *i*. After calculating MI_*ij*_ using Equation 4, we generate surrogates (which we subsequently use as a threshold for mutual information) by using a block-permutation procedure (Canolty et al., 2006): We simultaneously cut each time series into blocks of 1018 points (about 2 s), and we permute the resulting blocks uniformly at random. This procedure preserves lower frequencies and time-series features (such as nonstationarity and nonlinearity) below the chosen temporal scale. (See the Supplementary Information (Buldú & Porter, 2017) for further discussion and a detailed investigation of the influence of block length on the surrogates.) We then evaluate the mutual information between each surrogate time series to obtain MI^rand^.

### Frequency-Based Multilayer Networks

We construct a frequency-based multilayer network for each individual from the matrix that encodes the MI of each pair of sensors for the four different frequencies bands. Each layer includes nodes with the same frequency band, yielding four different layers: theta (*θ*), alpha (*α*), beta (*β*), and gamma (*γ*). We use MI^rand^ from the surrogate times series as a threshold and construct a weighted supra-adjacency matrix **W** with elements *W*_*ij*_ = MI_*ij*_ − MI_*ij*_^rand^ if MI_*ij*_ > MI_*ij*_^rand^ and *W*_*ij*_ = 0 otherwise. We thereby account only for edges with statistically-significant edges. (In Figure 2, one can see what fractions of edges are 0 in each case.) Finally, we apply a linear normalization to **W** to obtain

$$M_{ij}=\frac{W_{ij}-w_{\text{min}}}{w_{\text{max}}-w_{\text{min}}},$$

where *w*_min_ and *w*_max_ are, respectively, the largest and smallest entries of **W**. This ensures that *M*_*ij*_ ∈ [0, 1] for each individual, thereby facilitating comparisons between them. The weighted supra-adjacency matrix **M** includes some number of 0 entries, which account for interactions that we deem to not be statistically significant. It has four blocks along the diagonal that encode interactions within each layer (i.e., the same frequency band for different brain regions), and it has off-diagonal blocks that quantify coordination between different frequencies. See Figure 1B for a schematic.

### Methodological Considerations

An important issue is the applicability of our results to functional brain networks that are constructed using different approaches, such as by using source reconstruction or with different ways of evaluating coordination between brain regions. In our study, we constructed functional brain networks from magnetoencephalographic recordings, which are measured at the sensor level, instead of using the actual magnetic field that is generated in each brain region (i.e., at a source). (One can infer such a field using source-reconstruction methods.) Working with time series at the sensor level necessarily entails signal mixing (Schoffelen & Gross, 2009), whereas the use of source reconstruction has what is known as the “inverse problem” (of inferring the actual magnetic field that is created by the brain regions), and it thus requires the introduction of a priori assumptions in the model that one uses for source reconstruction. This issue has led to a diversity of algorithms to obtain source-reconstructed time series and, in many cases, different algorithms yield qualitatively different time series at the source level (see, e.g., Schoffelen & Gross, 2009; Belardinelli, Ortiz, Barnes, Noppeney, & Preissl, 2012). Consequently, there is an open debate about what constitutes the most appropriate methodology to construct functional networks using source reconstruction (Palva & Palva, 2012; van Diessen et al., 2015).

Regardless of how one constructs a functional brain network (and whether one uses sensors or sources), our analysis has the same qualitative implications. Specifically, estimating the value of *λ*_2_ from matrices associated with function brain networks is affected strongly by edge-weight heterogeneity and missing interlayer edges. One also observes the same qualitative differences between a multiplex construction and a full multilayer one, as this phenomenon depends on the number of interlayer edges and not on specific details of the construction of functional brain networks.

Similar reasoning applies to the construction of frequency-based brain networks using different ways of quantifying coordination between brain regions. We used MI because it is able to successfully capture both linear and nonlinear correlations, it is an adequate method for quantifying interdependencies between signals that are split into frequency bands (David, Cosmelli, & Friston, 2004), and it has been used widely in the past to construct functional brain networks (see, e.g., Bassett et al., 2009; Becker et al., 2012; Chai, Walther, Beck, & Fei-Fei, 2009; Deuker et al., 2009; Jin, Seol, Kim, & Chung, 2011). On the down side, MI cannot avoid zero-lag correlations that originate from common sources. (This flaw also occurs in many other commonly-employed similarity measures, such as partial correlation, coherence, and phase-locking value (Bastos & Schoffelen, 2016).) The systematic deletion of zero-lag correlations entails a very strict assumption, but because not all zero-lag correlations are due to common sources or volume conduction (Christodoulakis et al., 2013; Porz, Kiel, & Lehnertz, 2014; Vicente, Gollo, Mirasso, Fischer, & Pipa, 2008), there does not exist a consensus on how to eradicate them (see, e.g., Brookes, Woolrich, & Barnes, 2012; Stam, Nolte, & Daffertshofer, 2007). It is thus important for future work to conduct a systematic comparison between the most widespread coordination measures and their consequences on the spectral properties of functional networks. As we stated previously, the effect that edge-weight heterogeneity and missing interlayer edges have on functional multiplex and multilayer networks is a general phenomenon, as it does not rely on the specific similarity measure that one employs to evaluate coordination between brain regions, as long as there is sufficient heterogeneity in the weights of the interlayer edges. As shown in Brookes et al. (2016), such heterogeneity is expected in frequency-based functional brain networks, given the complicated interactions between different brain regions at different frequencies.

## AUTHOR CONTRIBUTIONS

Javier M. Buldú: Conceptualization; Data curation; Formal analysis; Methodology; Writing — original draft; Writing — revisions. Mason Alexander Porter: Conceptualization; Methodology; Writing — original draft; Writing — revisions.

## ACKNOWLEDGMENTS

We thank John Allen for fruitful conversations. We also thank the referees for their helpful suggestions.

J.M.B. acknowledges financial support from Spanish MINECO (project FIS2013-41057) and from Salvador de Madariaga Program (PRX15/00107), which allowed him to visit University of Oxford in summer 2016.

## FUNDING INFORMATION

Javier M. Buldú, Ministerio de Economía y Competitividad (http://dx.doi.org/10.13039/501100003329), Award IDs: FIS2013-41057 and FIS2017-84151-P. Javier M. Buldú, Ministerio de Economía y Competitividad (http://dx.doi.org/10.13039/501100003329), Award ID: PRX15/00107.

## Notes

1For such a matrix, *λ*_1_ = 0. See the subsection Heterogeneity and Missing Interlayer Edges in Multiplex Networks (in Results) for a detailed definition of the combinatorial supra-Laplacian matrix.2In general, networks that are constructed from pairwise time-series similarities have nonzero edge weights in all (or almost all) intralayer adjacency entries (Bassett & Sporns, 2017). However, in functional brain networks, the deletion of entries that are not statistically significant can lead to a non-negligible number missing edges, as is the case with our data.3It is also desirable, for example, to examine this type of network in a time-resolved manner (ideally using continuous time), to incorporate spatial constraints, and so on.4Analogous critiques are also relevant for research, including our own, on multilayer analysis of time-dependent brain networks (see, e.g., Bassett et al., 2011), for which one can envision incorporating time delays in coordination between brain regions.

## TECHNICAL TERMS

Multilayer brain network: A brain network with more than one layer. Layers are connected to each other through interlayer edges, which link node-layers from different layers.
Node-layer: Within each layer, a node-layer represents the dynamics recorded by a given magnetometer that is filtered at a specific frequency band. Thus, each magnetometer has an associated node-layer on each layer.
Layer: A portion of a multilayer network with a particular set of connections (called “intralayer edges”) between nodes. In our paper, each layer represents a specific frequency band of a functional brain network.
Multiplex brain network: A particular kind of multilayer brain network in which interlayer edges occur only between counterpart nodes (which, in this paper, represent the same brain region) in different layers.
Magnetoencephalography (MEG): Brain-imaging technique that measures the magnetic field of the brain using a set of magnetometers placed on the head of an individual.
Algebraic connectivity: The second-smallest eigenvalue (*λ*_2_) of the combinatorial Laplacian (or supra-Laplacian) matrix.
Combinatorial supra-Laplacian matrix: The combinatorial Laplacian matrix of a supra-adjacency matrix.
Cross-frequency coupling (CFC): In the brain, CFC is the mechanism through which dynamics at a certain frequency affect, or are affected by, the dynamics at other frequencies.
Combinatorial Laplacian matrix: Given the adjacency matrix *A* of a network and the degree matrix *D*, which consists of the degree of the nodes along the diagonal and *0*s in all other entries, the combinatorial Laplacian matrix is defined as *L* = *D* − *A*.
Mutual information (MI): A measure to evaluate statistical interdependencies between a pair variables, *X* and *Y*, based on comparing a joint distribution *p*(*X*, *Y*) with the product *p*(*X*)*p*(*Y*) of marginal distributions.
Supra-adjacency matrix: An adjacency matrix associated with a multilayer network; it codifies how much different node-layers interact with each other.
